# Assessing the Relationship Between the Flicker Test and Cognitive Performance

**DOI:** 10.3390/biology14111469

**Published:** 2025-10-22

**Authors:** Natalia D. Mankowska, Rita I. Sharma, Anna B. Marcinkowska, Jacek Kot, Pawel J. Winklewski

**Affiliations:** 1 Applied Cognitive Neuroscience Lab, Department of Neurophysiology, Neuropsychology and Neuroinformatics, Medical University of Gdansk, 80-210 Gdansk, Poland; anna.marcinkowska@gumed.edu.pl; 2 Department of Neurophysiology, Neuropsychology and Neuroinformatics, Medical University of Gdansk, 80-210 Gdansk, Poland; r.sharma@gumed.edu.pl (R.I.S.); pawelwinklewski@gumed.edu.pl (P.J.W.); 3 2nd Department of Radiology, Medical University of Gdansk, 80-210 Gdansk, Poland; 4 National Centre for Hyperbaric Medicine, Institute of Maritime and Tropical Medicine in Gdynia, Medical University of Gdansk, 81-519 Gdynia, Poland; jkot@gumed.edu.pl

**Keywords:** critical flicker fusion frequency, CFFF, flicker test, cognitive assessment, attention, reaction time, memory

## Abstract

**Simple Summary:**

Our study explored how a simple visual test, called the flicker test, relates to some cognitive processes in healthy adults. The flicker test measures how quickly a person can detect a light that appears to flicker on and off, which reflects brain alertness and information processing. We compared flicker test results with performance on memory and attention tasks, such as remembering sequences of numbers or blocks and responding quickly to visual cues. Our findings showed that some aspects of the flicker test vary slightly with age and gender but, overall, the results did not strongly predict cognitive performance in this healthy group. These results suggest that while the flicker test reflects certain aspects of brain function, it cannot yet replace standard cognitive tests. However, understanding how visual alertness relates to thinking may help in situations where people are exposed to demanding environments, such as diving, high-altitude work, or prolonged mental tasks. The study contributes to building knowledge about simple ways to monitor brain performance and could inform future research on maintaining cognitive function under challenging conditions.

**Abstract:**

An individual’s ability to process flickering light is expressed by critical flicker fusion frequency (CFFF), tested with the flicker test. CFFF is used to assess visual processing, arousal, and cognitive functioning, among other things, although it is unclear how it reflects these processes. Due to possible differences between CFFF values obtained in trials with increasing and decreasing frequency, it also remains questionable to use only averaged CFFF values in research. The main objective of the present study was to assess how CFFF is related to cognitive functions (attention, short-term and working memory, and executive functions), and psychomotor speed. The research objectives also included assessing the stability of CFFF and its variability with age and comparing CFFF between men and women. Thirty-six participants (17 women and 19 men) completed computerized cognitive tests (Simon and flanker tasks, the Corsi block-tapping task, and the digit span task) three times, along with the flicker test. We found that CFFF scores were stable across sessions but differed between fusion and flicker thresholds, with age significantly correlating only with the fusion frequency. Given that, we suggest that future studies analyze not only the averaged CFFF, but also examine flicker and fusion thresholds separately to better understand their distinct contributions. Our results also revealed generally weak correlations between CFFF and neuropsychological test scores, with significant associations found only in women, suggesting that CFFF may not be a reliable indicator of cognitive functioning.

## 1. Introduction

Critical flicker fusion frequency (CFFF) describes the frequency at which flickering light appears to be continuous. The upper boundary of visual processing abilities, called the CFFF threshold, defines the fastest flicker rate the visual system can perceive [1]. The results of CFFF testing can be influenced by many factors related to both the properties of the flashing light itself and subjects’ characteristics [1]. CFFF can be measured with what is known as a flicker test. Most commonly, it is used to measure an individual’s arousal and cognitive functioning, including functions like attention, information processing speed and reaction time, executive functions, and memory [2,3,4,5,6,7]. Flickering light has also been used by various researchers in different settings, e.g., [8,9,10,11,12]. However, it is still unclear if it provides an accurate assessment of cognitive functioning; until now, its relationship with traditional psychometric methods remains unclear. In the literature, statistical analyses of CFFF results are conducted in various ways. In some studies, the CFFF threshold is calculated as the average of trials with increasing (fusion) and decreasing (flicker) frequencies, e.g., [4,13,14]. Other studies distinguish between ascending and descending frequencies (e.g., [15,16,17]), while in some cases the CFFF is calculated based solely on either fusion or flicker trials [18,19,20]. It shows that researchers are not in agreement regarding a unified method of notation and calculation; however, studies suggest that the two types of frequency changes may be associated with somewhat distinct phenomena and processing mechanisms [15,17,21]. As we showed in previous study [22], the frequencies obtained in fusion and flicker trials differ significantly. Similar results were obtained by Muth et al. [23], who indicated that decreasing frequency was associated with higher CFFF values compared to trials with increasing frequency. Also, according to Haarlem et al. [24], the method of stimuli presentation can impact results and trials with ascending frequency may be somewhat more susceptible to producing outlier values. Thus, using only averaged values may not be justified, as this method may ignore important differences in flicker-fusion processing.

There are some sex differences in CFFF values, although they are not as pronounced as age-related differences. Some researchers suggest that these changes may be related to potential differences in how men and women process visual stimuli, which in turn may be the result of subtle physiological differences in the structure and function of the visual and nervous systems [25]. However, CFFF differences between men and women are not always observed. The characteristics of the study groups can sometimes make it difficult to directly compare results, as was the case in the Piispanen et al. [5] study, where a group of technical divers, most of whom were men, was analysed. The ambiguity of the results of previous studies complicates the formulation of clear conclusions.

Misiak [26], summarizing studies conducted among participants aged 5–80 years, reported that there is no clear change in CFFF from childhood to the teenage years, although there is a significant decrease in CFFF values after age 40. However, the same author, in his study of two equal (*n* = 50) age groups (19–30 and 63–87 years), noted there were significant CFFF differences between younger and older people, with older people achieving more varied results. At the same time, the groups of men and women he studied did not differ in their CFFF scores. In a more recent study conducted with a group of 1000 people aged 5–75 years, an increase in CFFF was observed up to the age of 16 by about 4 Hz per decade, after which CFFF values began to gradually decline [27]. Moreover, the decline in CFFF with age has been confirmed by subsequent researchers [23,28,29,30], especially around age of 60 and 70 years [23], suggesting a decline with age in the ability of the visual system to process rapidly changing visual stimuli [31]. Also, while the effect of aging on the eyes as a part of a visual system has been described [32], it is still not fully understood how stimuli like flashing lights are processed by the brain, in which, after all, the aging process also takes place [33]. The literature shows that aging has a significant impact on the processing of visual information, causing slower responses, reduced potential amplitudes, and differences between visual processing systems [33,34,35]. Regarding the latter, the motion processing system ages faster than the form processing system, which manifests itself in a greater delay in visually evoked potentials related to motion [33]. A broader understanding of this neurophysiological background could help in understanding to what extent CFFF changes are age-related. Thus, the main aim of this study was to assess and compare the outcomes of the flicker test with results from neuropsychological tasks among healthy subjects under normobaric conditions. Specifically, we sought to examine the correlation between the frequencies observed in flicker and fusion trials and their relationship with cognitive test performance. Additionally, with this study we aimed to evaluate the stability of the measurements obtained from both the flicker test and neuropsychological assessments, with a particular focus on their susceptibility to the learning effect. Because the association of CFFF with age is still not well understood, we also planned a targeted analysis to test this association. We were also interested in whether CFFF differs by gender. Although many of the studies to date did not indicate the existence of this relationship, we were keen to verify this in our study group.

We hypothesized that (1) CFFF correlates with cognitive test results; (2) there are differences in the frequencies obtained in flicker test trials with increasing and decreasing frequency; (3) CFFF does not change significantly in repeated trials performed on different days and is thus resistant to the learning effect; (4) CFFF decreases with age; and (5) CFFF does not differ significantly between men and women.

## 2. Methods

This study was conducted in the Department of Neurophysiology, Neuropsychology and Neuroinformatics and the Department of Hyperbaric Medicine and Maritime Rescue—National Center for Hyperbaric Medicine at the Medical University of Gdansk. Forty-seven people (25 women, 22 men) volunteered to participate in the study. Participants who provided incomplete data (*n* = 11, including 8 women and 3 men) were excluded from the analysis, resulting from dropouts in subsequent surveys. The final analysis included data from 36 subjects (17 women, 19 men) aged 19–46 years (men: M = 35.21, SD = 7.98; women: M = 29.24, SD = 7.64). All participants had normal or corrected-to-normal vision. Individuals were excluded if they met any of the following criteria: severe or uncontrolled medical or active conditions (neurological, psychiatric, cardiovascular, oncological diseases), pregnancy, or significant cognitive impairments that could hinder informed consent or study participation. Additionally, participants currently taking medications that could interfere with the study’s intervention were excluded. All subjects agreed freely and provided written informed consent. The study obtained the consent of the Bioethics Committee for Scientific Research at the Medical University of Gdansk, Poland (242/2020). Participants took the examination three times on different days and at different times of the day, between 9 a.m. and 9 p.m., so we performed a correlation analysis of the flicker tests’ results and hours to verify whether they were significantly related. There was no significant correlation between the hour of testing performed and the flicker test results on any subtests.

### 2.1. Flicker Test

In the flicker test we used a device flashing at 10–50 Hz with a frequency change rate 1 Hz/s, as we described in a previous study [22]. The diode of the flicker test device was 4 mm in diameter and was flickering with a light blue colour presented from about 30 cm. From each measurement, the data (frequency in Hz) were obtained on three parameters: fusion (increasing frequency), flicker (decreasing frequency), and the average of both, which is the CFFF value of the CFFF threshold.

### 2.2. Neuropsychological Assessment

In addition to the flicker test, the subjects performed a few neuropsychological tests, created online with the Psytoolkit software (version 3.4.6) [36,37]. They responded using a computer mouse. Each task was explained to them in detail beforehand and demonstrated in the trial version. To reduce the effect of learning, each trial was randomly generated, following the rules of the test. The entire neuropsychological examination took 10–15 min to complete, depending mainly on the maximum length of the sequences they reproduced in the selected tasks. The study used tasks involving various cognitive processes, as reported in Table 1. These tests were selected based on data from literature in which cognitive processes were compared with CFFF. A detailed description of each task is provided in the subsequent sections. Additionally, it was possible to obtain information on the number of correct and incorrect answers in each test.

#### 2.2.1. Digit Span Task

**Figure 1:**
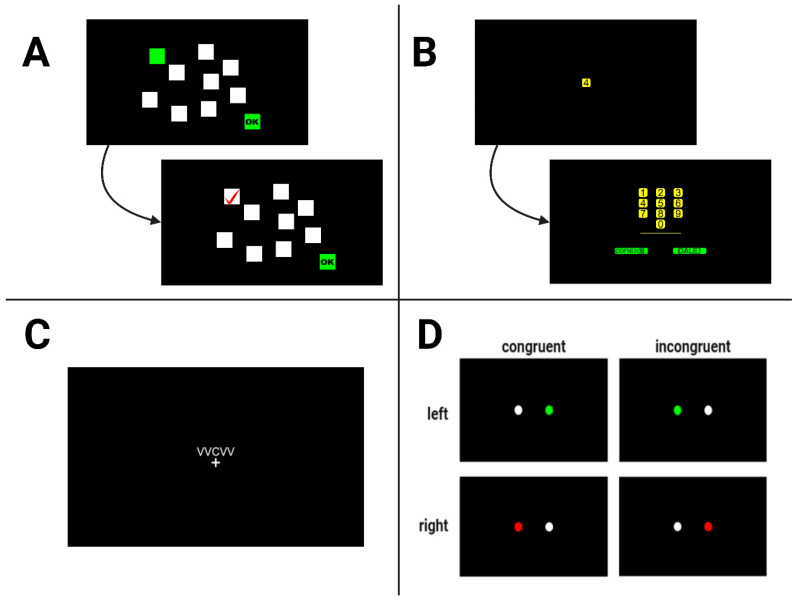
Simplified schematic flow of the (**A**) Corsi block-tapping task, (**B**) digit span task, (**C**) flanker task, and (**D**) Simon task (created with BioRender.com).

The original task was from the Wechsler’s Intelligence Test Battery (WAIS), where the examiner reads a sequence of digits at a rate of one per second [38]. The subject is required to recall sequences of digits in both the same (forward) and reverse order (backward) immediately after presentation. These two subtasks involve different memory processes: while the forward task mainly engages short-term memory, the backward task, due to the greater degree of manipulation of material in memory, involves more working memory. Successful performance on both tasks relies on executive functions, especially those involved in managing attentional processes. The length of the presented sequence increases with the length of correctly reproduced sequences (from two to nine items) until the participant fails two consecutive trials of the same digit length. The expected length of the reconstructed sequence should be in the range of 7 ± 2, according to the so-called Miller’s number, described as the number of elements a person can encode into short-term memory [39]. Typically, sequences reproduced in the backward direction are shorter than those reproduced in the forward direction [40]. Scores are calculated based on the longest sequence correctly recalled in each direction, expressed as a number from 2 to 9. Figure 1 shows a simplified flowchart of the task presented to the participants.

#### 2.2.2. Corsi Block-Tapping Task

This task is a visuospatial alternative to the Digit Span Task [38,41]. It assesses visuospatial short-term memory and working memory. In the original version, participants observe the examiner tapping a sequence of blocks and they are then asked to replicate the sequence in the same order (forward span) and in reverse order (backward span). The test starts with a sequence of two blocks, with the sequence length increasing until the participant fails to correctly reproduce two sequences of the same length. In contrast to the Digit Span task, differences between Corsi forward and backward recall are generally more difficult to detect [40]. In our study, participants were asked to observe a board with squares changing their colour from white to green in a random sequence, following the pattern shown in Figure 1. The positioning of the squares was in accordance with the coordinates described by Kessels et al. [42]. The primary measure is the longest sequence correctly reproduced, expressed as a number from 2 to 9.

#### 2.2.3. Simon Task

The Simon task evaluates cognitive control, particularly inhibitory control. Participants respond to the colour of stimuli presented on either the left or right side of a computer screen, with the location of the stimulus congruent or incongruent with the required response. The participants’ task was to use a computer mouse to press on the left side of the screen if the stimulus was green and on the right side if the stimulus was red, regardless of which side of the screen was actually displayed. The reaction times (RTs) in milliseconds and accuracy (correct, wrong, timeout) for all congruent and incongruent trials were recorded, with the Simon effect calculated as the difference in RTs between incongruent and congruent trials [38,43].

#### 2.2.4. Flanker Task

Flanker tasks have been used in cognitive psychology since 1974 as a method used to assess selective attention and inhibition [44]. Each participant was presented with a set of five letters on a screen, containing the letters V, B, X, and C. The participant’s task is to respond quickly enough to the letter target displayed in the centre of the screen, surrounded by other letter-distractors. The task involved two types of stimuli: congruent and incongruent. The congruent stimuli consisted of uniform sequences of characters (XXXXX, CCCCC, BBBBB, VVVVV). The incongruent stimuli featured sequences with a mix of characters, designed to create conflict between the central and surrounding characters (BB_BB, CC_CC, VV_VV, XX_XX, where “_” indicates a central character chosen from a set of mentioned four letters). Subjects were asked to answer using the mouse on the assigned side of the screen: left for letters X and C and right for letters B and V. Subsequent trials were randomly generated from the described sets (16 in total). The test measures the participant’s RT and accuracy at identifying the direction of the central stimulus, with conditions varying in the level of congruence between the central and flanking stimuli. The primary results are the RTs for congruent and incongruent trials and the differences in RTs (flanker effect, calculated as RT_incongruent_ − RT_congruent_) and accuracy between congruent and incongruent conditions.

### 2.3. Data Preparation

Both the flanker and Simon tasks yielded datasets of identical size, as all participants who completed the flanker task also completed the Simon task. Specifically, for each task, 2350 responses were collected in the first trial from 47 participants, and 1800 responses were collected in both the second and third trials from 36 participants, resulting in a total of 5950 responses per task (50 per participant). From the entire dataset for flanker task, incorrect responses (*n* = 379, 6.37%), late responses (*n* = 256, 4.30%), and those with a response time of less than 100 ms (*n* = 28, 0.47%) were filtered out. Thus, a final total of 5287 responses were included in the analyses of flanker test results (1830, 1699, 1758 for studies 1, 2, and 3, respectively). In the Simon task dataset, after excluding incorrect responses (*n* = 101, 1.07%) and late responses (*n* = 10, 0.17%), a total of 5839 responses were retained for further analysis: 2299 from the first trial, 1776 from the second, and 1764 from the third. This database did not include responses given faster than 100 ms.

After cleaning the database, the average RTs for the congruent and incongruent conditions were calculated for each participant in each task. From these, the flanker effect and Simon effect (RT in incongruent trials—RT in congruent trials) were computed, and these participant-level averages were then used for all subsequent analyses.

### 2.4. Statistical Analysis

Statistical analysis was performed with IBM’s SPSS Statistics 29.0.0.0 software. All numerical data were tested for normality with the Shapiro–Wilk test and Q-Q plots. Since the distributions of our data were generally heterogeneous, we decided to use non-parametric tests in the statistical analysis. For group comparisons, including repeated measures, we used Friedman test. The Wilcoxon signed rank test was used to compare pairwise. The Mann–Whitney U test was used to compare between independent groups (determined by participants’ gender). Correlation analysis was performed with the Spearman test. Statistical differences of *p* < 0.05 were considered significant. The figures were created in BioRender.com.

## 3. Results

### 3.1. Correlation Between Flicker Test and Neuropsychological Assessment Test Results

**Figure 2:**
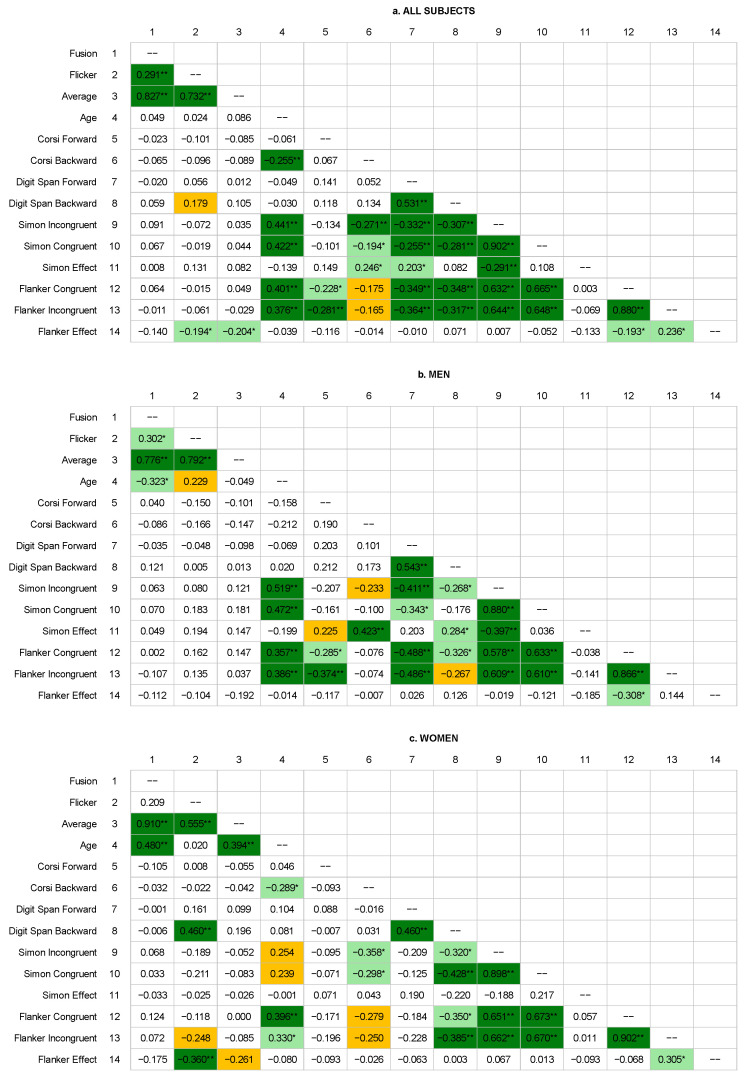
Correlation matrix of flicker test results (fusion, flicker, average), age, and neuropsychological test results. The values presented in the figure represent Spearman correlation coefficients (r). Dark green and ** denote correlations that are significant at the *p* < 0.001 level, while correlations at the *p* < 0.05 level are indicated in light green and *. Statistical trends (*p*-values close to 0.05) are marked in orange and described in the text.

The analysis of correlations between neuropsychological test scores and the frequencies of fusion, flicker, and average values suggested these were weakly correlated (see Figure 2). Among men (Figure 2b), no significant correlations were found (*p* > 0.05), while in the female group (Figure 2c), significant correlations were observed between flicker frequencies and the digit span backward scores (r = 0.460, *p* = 0.001) and the flanker effect (r = −0.360, *p* = 0.01). Some statistical trends for the group of women were observed in the correlation between average frequency and the flanker effect (r = −0.261, *p* = 0.064) and between flicker frequency and RTs in incongruent trials in the flanker task (r = −0.248, *p* = 0.079).

By comparison, we observed many statistically significant correlations between the individual neuropsychological test scores. In general, longer RTs were associated with shorter sequence lengths in the Corsi and digit span tasks. In both groups, the length of digit sequences recalled in the forward digit span task was moderately and positively correlated with the length of sequences recalled in the backward task (men: r = 0.543, *p* < 0.001; women: r = 0.460, *p* < 0.001; whole group: r = 0.531, *p* < 0.001). RTs in the Simon and flanker tasks were moderately correlated with each other (at the level of 0.58–0.67). The correlation matrix of data described above is presented in Figure 2.

Our analysis of correlations between flicker test values revealed significant differences in the relationships between the studied groups. In the analysis conducted across all subjects, statistically significant positive correlations were observed, including between fusion and flicker (r = 0.291, *p* = 0.002), fusion and average (r = 0.827, *p* < 0.001), and flicker and average (r = 0.732, *p* < 0.001) values. In the male group, these relationships were somewhat weaker but still highly significant: the correlation between fusion and flicker was 0.302 (*p* = 0.023) and between fusion and average it reached 0.776 (*p* < 0.001), comparable to the correlation between flicker and average (r = 0.792, *p* < 0.001). In contrast, among women, the correlation between fusion and average was even higher, at 0.910 (*p* < 0.001). A moderate and significant correlation was also found between flicker and average (r = 0.555, *p* < 0.001), while the relationship between fusion and flicker (r = 0.209) was not statistically significant (*p* = 0.142).

### 3.2. Measurement Stability

**Figure 3:**
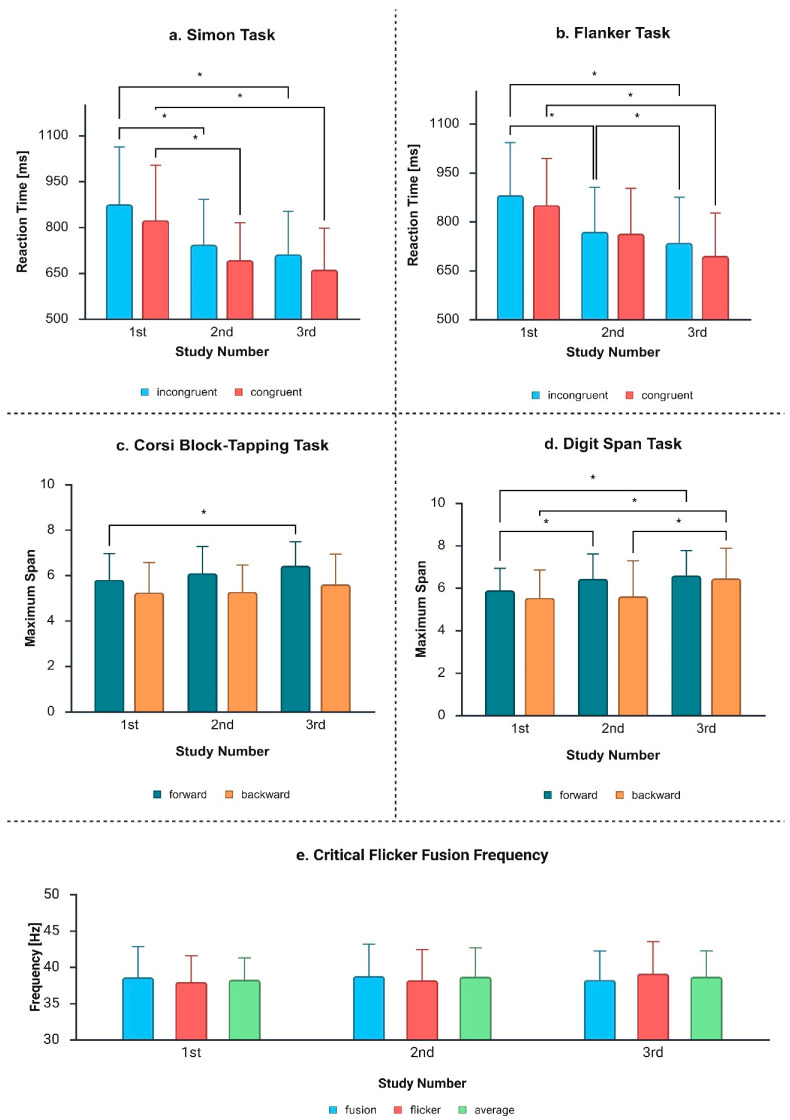
Repeated measurements of average reaction times in the (**a**) Simon task and (**b**) flanker task, divided into congruent and incongruent stimuli; (**c**,**d**) show the average lengths of the longest reproduced spans in the Corsi block-tapping task and digit span task, respectively; and (**e**) presents the repeated measurements of CFFF. Significant differences between the samples are marked with an asterisk (created with BioRender.com).

Because the subjects performed the tests three times, we analysed the stability of the measurements taken over time. Friedman test showed no statistically significant differences between the three measurements of flicker (chi^2^ = 2.722; df = 2; *p* = 0.256), fusion (chi^2^ = 0.722; df = 2; *p* = 0.697), and average (chi^2^ = 2.364; df = 2; *p* = 0.307) values. An additional pairwise (first-second, first-third, second-third) comparison showed no statistically significant differences between the fusion and average results, neither among men nor among women. Significant differences were found in the flicker subtest results between the first and third tests only among men (Z = −1.972, *p* = 0.049), which led to some statistical trend in average CFFF values in their group (Z = −1.751, *p* = 0.080). The flicker test results are presented in Figure 3e.

In the Simon task (see Figure 3a), mean RTs in incongruent trials were significantly higher than in congruent trials (first study: Z = −2.915, *p* = 0.004; second study: Z = −3.423, *p* < 0.001; third study: Z = −4.415, *p* < 0.001). The results appeared mixed in the flanker task (see Figure 3b), with statistical significance observed in the first (Z = −1.982, *p* = 0.047) and third study (Z = −3.252, *p* = 0.001), but not in the second study (Z = −0.557, *p* = 0.578). In flanker task, statistically significant differences between three trials were observed for both congruent (chi^2^ = 36.941; df = 2; *p* < 0.001) and incongruent conditions (chi^2^ = 39.588; df = 2; *p* < 0.001). Differences were obtained in the comparison of mean RTs between studies 1 and 2 and 1 and 3 (*p* < 0.001), but not between studies 2 and 3 (p_congruent_ = 0.088, p_incongruent_ = 0.083).

In the Flanker task, the group analysis showed significant differences between the mean RTs for each study, for both congruent (chi^2^ = 26.882, df = 2, *p* < 0.001) and incongruent trials (chi^2^ = 39.588, df = 2, *p* < 0.001). The results were confirmed by post hoc analysis (*p* < 0.001, except for 2–3 in the incongruent condition, where *p* = 0.027). The results are presented in Figure 3b. No statistically significant differences were observed in the Simon effect (chi^2^ = 0.412; df = 2; *p* = 0.814) and in the flanker effect (chi^2^ = 0.000; df = 2; *p* = 1.000).

Statistically significant differences were observed between the results of tests 1 and 3 in the Corsi block-tapping task forward (Z = −2.434, *p* = 0.015) and the digit span task forward (Z = −2.698, *p* = 0.007) and backward (Z = −3.118, *p* = 0.002). Additionally, results differed between studies 1 and 2 in the digit span task forward (Z = −2.557, *p* = 0.011) and between studies 2 and 3 in the backward subtest (Z = −3.140, *p* = 0.002). A graphical representation of the results from the Corsi Block-Tapping Task and the Digit Span Task is presented in Figure 3c and Figure 3d, respectively.

### 3.3. Sex and Age

The group of men and women we studied differed significantly in age (*p* = 0.028). We compared the results of both sexes to search for differences in the tests performed. We found sex differences in the flicker test results. The CFFF results with decreasing frequency (flicker) and CFFF threshold (average) values were slightly lower in men compared to women, but additional analysis showed that these differences were observed only in the first examination (flicker: U = 83.0, *p* = 0.013; average: U = 98.0, *p* = 0.044). The fusion results did not differ between women and men. The summary of data descriptives and statistical test values appears in Table 2.

**Figure 4:**
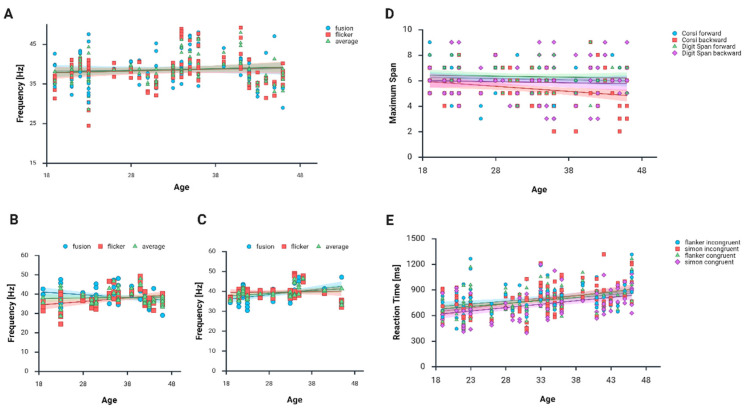
Scatterplots for age of participants and flicker test results among (**A**) all participants, (**B**) men, (**C**) women, (**D**) maximum length of reproduced span (for all subjects), and (**E**) reaction time (in milliseconds) in whole group (created with BioRender.com).

As shown in Figure 4B,C, fusion values correlated with age for both men (r = −0.323, *p* = 0.014) and women (r = 0.480, *p* < 0.001). Flicker frequencies were not correlated with age in any of these groups, although we observed a statistical trend across men (r = 0.229, *p* = 0.086), while in the group of women r = 0.020, *p* = 0.888. The CFFF threshold (average results) correlated with age in women (r = 0.394, *p* = 0.004) but not in men (r = −0.49, *p* = 0.717). Considering the whole group (see Figure 2a and Figure 4A), no correlation between flicker test results and age was observed (*p* < 0.05). See also Figure 2 for a summary of these results in a correlation matrix for all participants (Figure 2a), men (Figure 2b), and women (Figure 2c).

We found no significant differences between men and women in their neuropsychological test results (see Table 2 for descriptive statistics and the results of the comparative analysis). Therefore, Figure 4D,E present correlations between age and neuropsychological test scores for the entire group.

The maximum length of the span reproduced on the Corsi block-tapping task in the backward task correlated significantly with age only for women (r = −0.289, *p* = 0.044) but not for men (r = −0.212, *p* = 0.120). A similar relationship was not observed for the Corsi forward task (*p* > 0.05) or digit span results (all *p* > 0.05), as can be seen in Figure 2 and Figure 4D. As presented in Figure 2 and Figure 4E, there are some significant correlations of RTs and age. Specifically, there was a significant increase in RTs with age in men in the Simon task (incongruent: r = −0.519, *p* < 0.001; congruent: r = 0.472, *p* < 0.001), while for women these correlations in Simon task were nonsignificant (incongruent: r = 0.254, *p* = 0.073; congruent: r = 0.239, *p* = 0.091). Results of the flanker task performed by men also significantly correlate with age (incongruent: r = 0.386, *p* = 0.004, congruent: r = 0.357, *p* = 0.007), and unlike in the Simon task, significant correlations in the flanker task were also found for women (incongruent: 0.396, *p* = 0.004; congruent: r = 0.330, *p* = 0.018).

To verify whether these effects reflected stable relationships or were driven by interindividual variability and repeated-measures design, we additionally employed mixed linear models including age and sex as fixed effects and participant ID as a random factor. These models did not reveal any significant main or interaction effects of sex or age for any of the dependent measures, including CFFF, Corsi, Digit Span, Simon, and Flanker tasks. The only consistent influence was related to the order of testing, as described in previous section.

## 4. Discussion

In this study, we investigated the relationship between CFFF measured with flicker test results and neuropsychological test results. In our analysis, we also included comparisons of test variables with demographic factors (i.e., age, gender). The following section discusses the interpretation and implications of these findings.

### 4.1. CFFF & Neuropsychological Assessment

Although all the flicker test trials were significantly correlated with each other, differences in the strength of these correlations were apparent. While both flicker and fusion values correlated moderately or strongly with averaged results, the correlation between flicker and fusion was rather weak and did not occur in the female group. This may suggest some differences in the processing of flickering light in different directions of frequency change, especially among women. As described by Curran [45], it is possible that these differences are related to the so-called temporal adaptation to flicker, or they measure slightly different brain functions.

Several correlations between cognitive tests are noteworthy. Essentially, they indicated that the length of the reproduced spans in the Corsi block-tapping task and digit span task may be related to RT. Given that all these tasks require efficient attentional processes, one might think that they play an important role in moderating this relationship. Correlations between the RTs obtained in the Simon and flanker tasks were significant, but the strength of their relationship was moderate, which may suggest a different substrate of the processes involved in their performance.

In general, the results of the flicker test correlated poorly or not at all with the results of the neuropsychological tests. Moreover, unlike the flicker test, we did not find differences between men and women in their neuropsychological test scores, although we obtained slightly different correlational data from the two groups. We observed some correlations only in the women’s group, but only for the reproduction of digit sequences and the flanker effect. The direction of these correlations is interesting, especially in the context of the lack of correlation between flicker and fusion values among women. It seems that as flicker thresholds increased, women performed better in terms of working memory of verbal material, and at the same time, they showed an improved ability to control reactions in conflict situations. It is not entirely clear why.

Mewborn et al. [46] tested whether higher CFFF scores are associated with better performance on cognitive tests. Their study confirmed this relationship only for executive function (including set shifting, updating, inhibition), as measured by the shifting attention task from the CNS-Vital Signs computerized cognitive battery. They showed that CFFF was a good predictor of executive functioning, regardless of age (they studied two groups: 72 younger adults with an average age of 21.7 years, and 57 older adults with a mean age of 72.4 years). At the same time, the authors did not confirm that CFFF could well predict other cognitive parameters (verbal memory, visual memory, processing speed, and reasoning) and global cognition (measured by a Clinical Dementia Rating only in the older group). Similarly, Jensen [47] noted that it is safe to assume the absence of or a weak association of CFFF with intelligence (understood as Spearman’s g factor, measured by him with Advanced Progressive Matrices and Terman’s Concept Mastery Test). Given that executive function performance is dependent on sensory function and information processing speed [48,49], our observations appear consistent with these results per the relationship between flicker thresholds and working memory performance and response inhibition in conflict situations. As Mewborn et al. [46] pointed out, “a fast and efficient brain would also have improved executive function” (see Discussion section).

According to a study by Curran and Wattis [16], descending thresholds (flicker) correlated with some tests (choice reaction time, Abbreviated Mental Test, Global Deterioration Scale and Rey Auditory Verbal Learning Test) yet at the same time were not correlated with the results of other tests (Mini-Mental State Examination, Sandoz Clinical Assessment Geriatric Scale, digit span forward and backward, Trail Making Test and word fluency). In addition, descending thresholds were not significantly correlated on the digit symbol substitution test, although they correlated with averaging and ascending (fusion) frequencies. According to these authors, CFFF may reflect cortical function among people with Alzheimer’s disease, although they noted that caution is needed about exactly which cognitive parameters are used in the inference process. The results of our study do not clearly justify the use of CFFF as an indicator of cognitive functioning. Additional research is needed to better clarify the relationship between CFFF and cognitive functioning. Nevertheless, considering the potential applications of these findings, it is important to note that the relationship between CFFF and cognitive functioning may hold practical relevance beyond laboratory settings. In operational environments or under specific physiological conditions—such as hypoxia, hyperbaric exposure, diving, fatigue, or exposure to stressors—subtle changes in cortical arousal and attentional control can critically influence performance efficiency and safety [3,22,50]. In this context, CFFF could serve as a rapid, non-invasive indicator of temporary cognitive or neurophysiological alterations, supporting its potential use in monitoring alertness, fatigue, or information-processing capacity in real-world conditions (e.g., diving, aviation, or spaceflight). Further studies combining CFFF measurements with cognitive and behavioral indices in such conditions could therefore help to clarify whether the relationships observed under laboratory conditions translate into functionally meaningful outcomes in applied settings.

### 4.2. Measurement Stability

Our results indicate high stability in CFFF measurements over time. These results are consistent with other studies on the subject. For example, Jensen [47] obtained an average measurement reliability of r = 0.941, while other researchers found a reliability of r = 0.903 [16]. Still, Curran and Wattis [16] observed slightly higher stability for descending threshold compared to ascending threshold (r = 0.943 compared to 0.840). What is puzzling, however, is the result in samples with decreasing frequency (flicker) among men, which indicated differences between the first and third measurements. Although statistical analysis has indicated that these differences are significant, they were at the limit of significance (*p* = 0.049). Perhaps this result is related to the method of limits we used in the flicker test, which Eisen-Enosh et al. [9] wrote about as showing lower repeatability. However, compared to other methods (method of constant stimuli or the staircase method), the limit method requires significantly less time to perform the test (up to 11 times less), which minimizes the fatigue of subjects, which, as it turns out, has a significant impact on results obtained [51,52]. As Landis [53] pointed out, it is difficult to assess whether there is any physiological basis for CFFF changes. Although his work is decades old, his observation that in the case of CFFF, the so-called practice effect could be related to a change in subjective decision of what a flickering light looks like as its frequency increases or decreases is noteworthy. He emphasized:

More than half of observers shift this standard upward to higher F’s [frequencies] during the first three or four periods of observation. The remaining observers either shift to a lower standard or show no consistent trend to shift either up or down [53] (Attitudes; Practice; Criteria section).

Conversely, others have observed an increase in CFFF in the second and third trials compared to the first [28]. Considering the lack of differences between the fusion and average scores among men and women, one might suspect that the slight increase in flicker frequencies in men may be related to the interaction of several factors, probably age-related.

In our group, the greatest change in RTs occurred between the first and second trials, which may reflect a learning effect [54,55]. Interestingly, the changes between the second and third trials were not as pronounced in the results obtained in the Simon task, although they become apparent in the flanker task. However, these changes were visible only in the congruent samples, while in the incongruent samples they were not pronounced enough to reach statistical significance. However, due to the existence of a statistical trend (*p* = 0.074), they cannot be excluded. These differences between the Simon and flanker tasks may suggest that the Simon task may be more susceptible to the learning effect, as it requires the processing of non-verbal stimuli and providing answers based on the direction or location of the data presentation, while in the flanker task stimuli are presented in the same place and require consideration of more complex information. We did not observe the Simon or flanker effects changing over time. Welch and Seitz [56] noted that the Simon effect can be reduced with training (while not a permanent change), and this effect can be observed again after a break. This suggests that the suppression of irrelevant information is an active process which requires good cognitive control. It should be noted, however, that in their study, the Simon task was performed daily by the participants, and they had a 1 min break after every 48 trials (in total, 960 trials per day), while in our study the intervals between trials were days or even weeks. Therefore, perhaps the intervals between our studies were long enough to reduce the impact of training.

We observed that the maximum length of the reproduced spans increased a little, and these changes were significant. Again, this may be related to the learning effect [57]. Unlike the Corsi block-tapping task, the digit span task seems to be more susceptible to the learning effect, which may be related to the ability to verbalize the information processed, which helps to sustain it in short-term memory.

### 4.3. Sex and Age

In our study, only flicker scores differed by gender, with the group of women scoring slightly higher than men. This represents an inverse relationship compared to the study of Bernardi et al. [28], which noted that men tended to score slightly higher on the flicker test than women, particularly in specific areas of the visual field. Also, Venevtseva et al. [52] found that fusion and average frequencies are higher in males than females with no differences in flicker frequencies. These examples show that it is possible that the relationships between CFFF thresholds and gender are moderated by additional factors, which is supported by others (see, for example, literature reviews such as [1,58]). Our data showed a correlation between age and CFFF thresholds in trials with decreasing frequency (flicker) in men, while no such relationship was observed in women. In trials with increasing frequency (fusion), the results of men and women were similar and significantly correlated with age. Also, the CFFF threshold correlated with age in women but not in men. It should be noted, however, that the group of men we studied was slightly older than the group of women (men: M = 35.02; women: M = 28.92), although the age range differed slightly (participants over 18 years old and up to 47 for men and up to 45 for women). Nevertheless, fusion thresholds have been suspected to be more sensitive to age-related changes among both men and women. However, not all studies have confirmed a decrease in CFFF values with age. For example, Mewborn et al. [46] conducted a study with a group of younger (M = 21.7 years) and older (M = 72.4 years) people, finding no interactions between CFFF and age group. Curran and Wattis [16], on the other hand, found no correlation of CFFF measures with age, although they emphasized that the difference between frequencies achieved in decreasing and increasing frequency trials increased with age in their group.

It is important to note here the discrepancies between individual CFFF parameters, that is, between trials performed with increasing or decreasing frequency and average values. Guided by the conclusions of previous studies, it is possible that these trials may reflect slightly different aspects of visual system performance [45,59]. For example, Curran and Wattis [16] revealed that flicker results were significantly higher than fusion frequencies, and these results were consistent throughout their studies [16,21,45,59]. None of their results (fusion, flicker, average) were correlated with age, although they found a correlation between age and the difference between fusion and flicker results. Granted, their group was significantly older than ours (M = 71.3, SD = 9.97, age range: 60–91 years), so this makes it difficult to infer the dynamics of CFFF changes, although it is possible that in this age group CFFF changes are no longer as pronounced as in younger individuals. What is interesting, however, is the difference between samples per increasing and decreasing frequency, indicating the validity of separating them in the analyses. There is a possibility, however, that changes in the frequency range obtained in flicker and fusion trials may be correlated with various physiological and psychological processes. Moreover, some studies have indicated that in groups of people with an “uncertain” approach to the test, no differences are observed between ascending and descending thresholds, while in groups of “reckless” people, descending values are significantly higher than ascending ones, which may suggest a link with personality traits [60,61]. In addition, Curran and Wattis [16] found that in a group of healthy elderly people, frequencies obtained in decreasing-frequency (flicker) trials were higher than results in increasing-frequency (fusion) trials—an inverse relationship compared to their Alzheimer’s disease group. The authors hypothesized that these differences may be a sign of pathological disease processes in Alzheimer’s [16,59]. For this reason, we recommend considering each of these parameters separately (and not just as an average value), as they did.

The results of the cognitive tasks indicated several key findings related to age and performance differences between men and women. The Corsi block-tapping task backward showed a significant negative correlation with age for both men and women, suggesting that as age increases, performance on this task declines. However, no significant age-related correlations were found in the Corsi block-tapping task forward or the digit span task, indicating that the Corsi block-tapping task backward may be more sensitive to age-related decline, as it engages more cognitive load.

The significant differences we observed between the RT lengths in the Simon and flanker tasks indicate that participants took significantly longer to respond in the incongruent trials compared to the congruent ones. These results are consistent with the literature on the subject, stating that it takes longer to make a decision in a conflicting situation than when receiving consistent information [17,44,62]. As in other studies, RTs exhibited a strong overall correlation with age, highlighting a clear trend of slower responses as participants aged [43,63]. Unlike Overbye et al. [63], the flanker effects we obtained did not correlate with age, but their study group was much younger than ours and included children and adolescents aged 8–19 years. Their observations indicate that RTs were significantly shorter in adolescents compared to younger individuals. Considering our results, it can be concluded that RTs improve up to a certain point, only to lengthen again during aging. Although no such correlation has been observed in women, in the Simon task, men showed significant positive correlations between age and performance in both incongruent and congruent conditions, implying that older men might perform differently than younger men, potentially indicating increased difficulty with age in the processing of conflicting information. Finally, the flanker task revealed significant correlations for both genders in both incongruent and congruent conditions, with stronger effects observed in males, suggesting that men may experience more pronounced age-related changes in executive control tasks. These findings confirm that age affects cognitive performance differently across tasks and genders, with some tasks showing more sensitivity to age than others.

### 4.4. Limitations

We found no correlation between the hours of testing and the frequencies obtained in the flicker test. This may suggest that CFFF levels are relatively constant throughout the day, so we excluded these changes as a possible moderating factor in our results. However, we did not test how much the CFFF measurements changed throughout the day for each participant. This could be an interesting direction, however, given the research on the effect of fatigue on CFFF results [51,64]. In addition, the ages of female and male participants were slightly different. Nevertheless, we suspect that this was of little significance in our study, given reports in the literature that differences begin to become apparent after about the age of 40 years [26].

## 5. Conclusions

Our neuropsychological test results showed greater variability in results over time compared to the flicker test, which was relatively stable over time when performed under similar conditions. We confirmed the independence of the flicker test from the learning process.

Both the results of “classic” cognitive tasks and the results of the flicker test showed age-dependent variability. However, we did not find a clear and strong relationship between CFFF and cognitive test scores, which raises questions about whether the flicker test is a good measure of cognitive function. Future research should clarify these inconsistencies.

Although our results were inconclusive, the lack of consistency between fusion and flicker thresholds suggests that they may reflect different aspects of visual processing. We therefore recommend analyzing these measures separately in future studies, as relying solely on the averaged CFFF threshold may obscure meaningful differences. Disaggregating fusion and flicker data could help clarify whether—and how—these components differ, which may be essential for advancing our understanding of temporal visual processing.

## Figures and Tables

**Table 1:** Cognitive functions involved in the performance of neuropsychological tests.

Neuropsychological Test		Reaction Time	Attention	Inhibition	Working Memory	Short-Term Memory
Corsi block-tapping task	Forward	−	+	−	−	+ (visual)
	Backward	−	+	−	+ (reversing sequence)	−
Digit span task	Forward	−	+	−	−	+ (verbal)
	Backward	−	+	−	+ (reversing digits)	−
Simon task		+	+	+ (inhibit automatic response)	−	−
Flanker task		+	+	+ (suppress distractor response)	−	−

“+” indicates that the function is measured by the task, “−” that it is not.

**Table 2:** Comparison of age and individual test scores between men and women.

		Men				Women				U	*p*
		*n*	M	Me	SD	*n*	M	Me	SD		
Age		19	35.21	36.00	7.98	17	29.24	29.00	7.64	832.5	0.000 *
CFFF	Fusion	57	38.51	37.72	4.17	51	38.52	37.95	4.24	1449.0	0.978
	Flicker	57	37.39	37.48	4.41	51	39.54	39.21	3.39	1006.5	0.006 *
	Average	57	38.12	37.62	3.72	51	39.00	38.53	3.20	1215.5	0.143
Corsi block-tapping task	Forward	55	6.24	6.00	1.17	51	6.00	6.00	1.13	1310.0	0.545
	Backward	55	5.35	5.00	1.34	49	5.41	5.00	1.22	1326.5	0.888
Digit span	Forward	55	6.15	6.00	1.18	51	6.49	6.00	1.12	1214.0	0.216
	Backward	54	5.76	6.00	1.68	51	5.98	6.00	1.35	1261.5	0.446
Simon task	Incongruent	55	805.67	765.85	181.89	51	743.24	778.40	159.73	1131.0	0.086
	Congruent	55	746.90	730.96	162.48	51	701.13	714.71	159.70	1161.0	0.127
	Simon effect	55	−58.77	−42.25	81.25	51	−42.11	−42.12	69.25	1244.0	0.316
Flanker task	Incongruent	55	805.36	775.96	148.31	51	782.42	763.09	167.65	1302.0	0.525
	Congruent	55	784.93	766.83	155.62	51	751.05	727.35	144.96	1219.0	0.246
	Flanker Effect	55	20.43	23.12	76.92	51	31.37	29.04	73.83	1335.0	0.670

*n*= number of included samples, M = mean, Me = median, SD = standard deviation, U = Mann–Whitney U test, *p* = *p*-value. The *n* values take into account the results from all three trials in which the participants took part, so they were larger than the size of the group we studied (*n* = 36). An asterisk (*) indicates statistically significant values.

## Data Availability

The raw data that support the findings of this study are not openly available due to reasons of sensitivity. The processed dataset is included in the Supplementary Materials. None of the experiments were preregistered. Statistical analysis was performed with IBM’s SPSS Statistics 29.0.0.0 software. Analysis code is available in the Supplementary Material.

## Supplementary Materials

The following supporting information can be downloaded at: https://www.mdpi.com/article/10.3390/biology14111469/s1. The Supplementary Materials contain two versions of the processed dataset—one in long format and one in wide format—both representing the same underlying data. In addition, files describing the variables and the SPSS syntax used for the analyses are included.
